# Molecular docking and biochemical validation of (-)-syringaresinol-4-O-β-D-apiofuranosyl-(1→2)-β-D-glucopyranoside binding to an allosteric site in monoamine transporters

**DOI:** 10.3389/fphar.2022.1018473

**Published:** 2022-10-28

**Authors:** Hanhe Liu, Yingyao Wu, Chan Li, Qingfa Tang, Yuan-Wei Zhang

**Affiliations:** ^1^ School of Life Sciences, Guangzhou University, Guangzhou, China; ^2^ School of Traditional Chinese Medicine, Southern Medical University, Guangzhou, China; ^3^ Guangdong Provincial Key Laboratory of Chinese Medicine Pharmaceutics, Guangzhou, China

**Keywords:** *Albizia julibrissin*, antidepressants, mechanism of action, serotonin transporter, monoamine transporters, molecular docking, allosteric site, (-)-syringaresinol-4-O-β-Dapiofuranosyl-(1→2)-β-D-glucopyranoside

## Abstract

*Albizia julibrissin* Durazz is one of the most common herbs used for depression and anxiety treatment, but its mechanism of action as an antidepressant or anxiolytic drug have not been fully understood. We previously isolated and identified one lignan glycoside compound from *Albizia Julibrissin* Durazz, (-)-syringaresinol-4-O-β-D-apiofuranosyl-(1→2)-β-D-glucopyranoside (SAG), that inhibited all three monoamine transporters with a mechanism of action different from that of the conventional antidepressants. In this study, we generated homology models for human dopamine transporter and human norepinephrine transporter, based on the X-ray structure of *Drosophila* dopamine transporter, and conducted the molecular docking of SAG to all three human monoamine transporters. Our computational results indicated that SAG binds to an allosteric site (S2) that has been demonstrated to be formed by an aromatic pocket positioned in the scaffold domain in the extracellular vestibule connected to the central site (S1) in these monoamine transporters. In addition, we demonstrated that SAG stabilizes a conformation of serotonin transporter with both the extracellular and cytoplasmic pathways closed. Furthermore, we performed mutagenesis of the residues in both the allosteric and orthosteric sites to biochemically validate SAG binding in all three monoamine transporters. Our results are consistent with the molecular docking calculation and support the association of SAG with the allosteric site. We expect that this herbal molecule could become a lead compound for the development of new therapeutic agents with a novel mechanism of action.

## Introduction

*Albizia julibrissin* Durazz is one of the most popular herbs that has been historically used for depression and anxiety treatment in traditional Chinese medicine. The major ingredients in *Albizia Julibrissin* Durazz include triterpenoids, lignans, flavonoids, saponins, and sterols (Cai et al., 2015; Wang et al., 2019; Li and Yang, 2020; Lu et al., 2020; Li R. et al., 2022b). Preclinical studies have demonstrated that the ingredients isolated from its dried flowers or bark exhibited multiple pharmacological properties including antidepressant and anxiolytic activities (Kang et al., 1999; Jung et al., 2013; Liu et al., 2017). Despite significant progress in characterizing bioactive constituents and their pharmacological roles, the molecular mechanism of action of *Albizia julibrissin* Durazz as an antidepressant or anxiolytic drug remains to be understood.

Monoamine neurotransmitter transporters are transmembrane proteins located at presynaptic plasma membrane of monoaminergic neurons, including serotonin transporter (SERT), dopamine transporter (DAT), and norepinephrine transporter (NET). They terminate monoaminergic signaling by the reuptake of monoamine neurotransmitters from the synaptic cleft in the central nerve system (Legakis et al., 2020; Docherty and Alsufyani, 2021). Among these monoamine transporters, SERT is of particular interest because it is the principal target for antidepressant drugs, such as selective serotonin reuptake inhibitors (SSRIs) that are commonly used for depression treatment in clinical practice. Serotonin-norepinephrine reuptake inhibitors (SNRIs), such as duloxetine (Chow and Issa, 2017; Robinson et al., 2022) and venlafaxine (Suwala et al., 2019; Smolarczyk-Kosowska et al., 2022) that simultaneously inhibit both SERT and NET were also approved as antidepressant or anxiolytic drugs. However, none of triple reuptake inhibitors (TRIs) that concomitantly inhibit SERT, DAT, and NET is currently available in the market (Davis et al., 1997; Laizure and Cyr, 2000; Barbey, 2002).

The previously resolved high-resolution structures of DAT and SERT bound with their specific inhibitors have provided structural insights into the molecular basis for antidepressant action on the monoamine transporters (Penmatsa et al., 2013; Penmatsa et al., 2015; Coleman et al., 2016; Coleman et al., 2019). In these structures, antidepressant molecules have been shown to occupy the central binding site (S1) and thus to competitively inhibit monoamine neurotransmitter transport across plasma membrane. Strikingly, an allosteric site (the second substrate or ligand binding site, S2) has been recently revealed to be formed by an aromatic pocket positioned in the scaffold domain in the extracellular vestibule connected to the central binding site in the cryogenic electron microscopy (cryo-EM) and crystal structures of SERT (Coleman et al., 2016; Yang and Gouaux, 2021). Additionally, a non-competitive inhibitor, vilazodone, has been demonstrated to bind to the S2 site in SERT by structural and pharmacological approaches (Plenge et al., 2021). These works have shifted our efforts in developing antidepressants toward novel agents that target the S2 site in monoamine transporters.

We have recently isolated and identified two lignan glucosides, (-)-syringaresinol-4-O-D-apiofuranosyl-(1→2)-D-glucopyranoside (SAG) and (-)-syringaresinol-4,4′-bis-O-D-glucopyranoside (SBG), from the bark of *Albizia julibrissin* Durazz that acted on SERT by a novel underlying mechanism of action different from that of SSRIs (Huang et al., 2022). Our previous results showed that the two lignan glycosides noncompetitively inhibited SERT activity and also induced a conformational shift toward an outward-closed state of the transporter protein. Thus, these compounds were proposed to directly bind to the transporter protein, presumably to the S2 site, thereby, to noncompetitively inhibit SERT activity by blocking essential conformational conversion for substrate transport (Huang et al., 2022). Of these compounds, SAG is of special concern to study further because it has been demonstrated to exert an antidepressant and anxiolytic activity in acute restraint-stressed rat models in an earlier study, its molecular mechanism of action, however, was not uncovered (Liu et al., 2017).

In the present work, we conducted the molecular docking of SAG to the S2 site in the structure of human SERT (hSERT) and homology models of human DAT (hDAT) and human NET (hNET). In addition, we investigated the effect of SAG on conformation of the cytoplasmic pathway in SERT. Furthermore, we performed mutagenesis of the residues in the allosteric and orthosteric binding sites to biochemically validate SAG binding in all three monoamine transporters. Our results provided new insights into the association of SAG with the S2 site and also the specificity of SAG inhibition of monoamine transporters.

## Materials and methods

### Materials

(-)-Syringaresinol-4-O-β-D-apiofuranosyl-(1→2)-β-D-glucopyranoside (SAG) was purchased from Chengdu HerbSubstance Biotechnology Co. Ltd., Sichuan, China (batch no. PCS0316). HeLa cells (CCL-2) were from American Type Culture Collection. The expression plasmids for hSERT, hDAT, and hNET were from the Dr. Rudnick lab (Yale School of Medicine). The S277C/X5C expression plasmid used in this study was described previously (Rudnick, 2006). 4-[4-(Dimethylamino)phenyl]-1-methylpyridinium (APP^+^), 4-[4-(dimethylamino)styryl]-N-methylpyridinium (ASP^+^), fluoxetine, GBR12909, and desipramine were purchased from Sigma-Aldrich. Lipofectamine 2000 and Micro BCA protein assay reagent kit were obtained from Thermo Fisher Scientific. 2-Aminoethyl methanethiosulfonate hydrobromide (MTSEA) was purchased from Biotium. All other reagents were of analytical grade.

### Homology modeling of human DAT and human NET

Homology models of hDAT and hNET were generated with Modeller (Webb and Sali, 2016) based on an outward-facing structure (2.89 Å resolution, PDB ID 4XP1) of the *Drosophila melanogaster* DAT (dDAT). The alignment between two sequences was obtained with ClustalW and covered residues 58 to 198 and 209 to 601 for hDAT or 54 to 187 and 199 to 598 for hNET, that is, all except a portion of extracellular loop 2 and the N and C termini. The sequence identity between the target and the template is 52% for hDAT or 55% for hNET, respectively. The models with the highest scores were selected for visualization. Figures of structures and models were generated using PyMOL v2.5.2 (Schrödinger, Inc.).

### Molecular docking

Molecular docking was carried out with Glide software in Schrödinger Suites v2021.2 on the structure of hSERT (PDB ID, 7LIA, 3.5 Å) and the models for hDAT and hNET. The 15B8 Fab (chains B and C) and ligands such as 5-HT, acetylglucosamine, cholesterol, pentane, heptane, decane, and dodecane present in the hSERT structure were removed. The template structure was then subjected to automated structure preparation using the Protein Preparation Wizard in order to optimize the hydrogen bonding network, conformation of bonds, and energy constraints. Ligand preparation was preformed using Ligprep. The SAG molecule was input into Maestro and optimized for its conformation and energy in the OPLS4 force field (Lu et al., 2021). Protonation states of the ligand were calculated using Epik at pH 7.0 ± 2.0. Docking was performed using the Glide module of Schrödinger under a standard precision with the ligand in a flexible conformation. In the docking step, 20 poses for SAG in each transporter protein were generated using Van der Waals radius scaling of 0.8 for the protein and ligand. The ligand posing with the S2 residues within 5 Å was subject to conformational search and energy minimization. The refined hSERT-SAG, hDAT-SAG or hNET-SAG complex was ranked by glide scores, respectively. The more negative the glide score was, the more favorable the ligand binding to the S2 residues. One pose with the lowest energy for each transporter was exported into PyMOL for visualization.

### Site-directed mutagenesis

The mutants used for this study were constructed in the WT background carried by the expression plasmids for hSERT, hDAT, or hNET under the control of CMV promotor in pcDNA3.1 vector, respectively. All mutants were generated using the Mut Express II Fast Mutagenesis Kit (Vazyme) and confirmed by full-length DNA sequencing.

### Expression of human SERT, human DAT, and human NET

HeLa cells were cultured in Dulbecco’s Modified Eagle’s Medium supplemented with 10% fetal bovine serum, 100 units/ml penicillin, and 100 μg/ml streptomycin at 37°C in a humidified 5% CO_2_ incubator. Cells were plated in a 6-well culture plate and grown overnight. Cells at ∼70% confluence were transfected with hSERT, hDAT, or hNET cDNA in pcDNA3.1 by lipofectamine 2000. Transfected cells were incubated for 24 h at 37°C with 5% CO_2_ and then transferred into a 12-well plate placed with polylysine coated slides. After grown for additional 12–16 h, the cells were assayed for APP^+^ or ASP^+^ fluorescence image acquisition.

### APP^+^ uptake and ASP^+^ binding measurements

The cells expressing WT or mutants of hSERT, hDAT, or hNET were wet mounted on polylysine coated glass slides in 12-well plates and applied for the indicated treatments. In brief, the cells were washed twice with 500 μl KRH buffer containing 20 mM HEPES, pH 7.4, 120 mM NaCl, 1.3 mM KCl, 2.2 mM CaCl_2_, 1.2 mM MgSO_4_, and 0.1% (w/v) glucose. APP^+^ uptake was measured by adding 500 μl KRH buffer containing 2 μM APP^+^ and incubating for 5 min at room temperature. Excess APP^+^ was then removed by rapid washing three times with 500 μl KRH buffer. The extent of APP^+^ accumulated in the cells was determined by confocal imaging analysis. ASP^+^ binding to SERT in the cell membrane was measured with digitonin-permeabilized cells (Li M. et al., 2022a). The cells expressing WT or mutants of hSERT were incubated with 10 μM ASP^+^ in the presence of 25 μg/ml digitonin at room temperature for 5 min. After removing excess ASP^+^ by rapid washing three times, ASP^+^ binding was determined by confocal imaging analysis. Nonspecific APP^+^ transport or ASP^+^ binding was measured in the presence of 10 μM fluoxetine, GBR12909, or desipramine and subtracted to give APP^+^ uptake or ASP^+^ binding, respectively.

### Cytoplasmic cystine accessibility measurements

Accessibility measurements were performed with the cells expressing S277C/X5C (Li M. et al., 2022a). The cells were treated with 0.2 mM MTSEA in KRH buffer containing the indicated ligands in the presence of 25 μg/ml digitonin at room temperature for 5 min. The cells, then, were washed free of unreacted MTSEA and ligands, ASP^+^ binding was measured by adding 500 ul KRH buffer containing 10 μM ASP^+^ and incubating at room temperature for 5 min. Excess ASP^+^ was removed by ×3 rapid washing with KRH buffer and ASP^+^ fluorescence retained in the cell membrane was measured by confocal imaging analysis in the digitonin-permeabilized cells. Non-specific ASP^+^ binding was measured by adding 10 μM fluoxetine.

### Fluorescence image acquisition and fluorescence intensity analysis

Images were acquired at room temperature using a 20 or ×60 water immersion objective with the Zeiss LSM 900 confocal microscope (Li M. et al., 2022a). APP^+^ and ASP^+^ were excited by an argon laser with excitation peak at 488 nm. A field of cells at an appropriate density was selected and APP^+^ or ASP^+^ fluorescence was captured. Images were analyzed using Zen Blue software. Fluorescence intensity was counted and normalized to the cell or cell membrane areas (mean fluorescence).

### Data analysis

Nonlinear regression fits of experimental and calculated data were performed with Origin (Origin Lab). The statistical analysis given was from multiple experiments. Data with error bars in the figures represent the mean ± SD for at least 10 measurements per condition in one experiment or the mean ± SEM from at least three experiments as indicated, respectively. Statistical analysis was performed using One-Way ANOVA under SPSS 26.0.

## Results

### Modeling of human DAT and human NET

**FIGURE 1:**
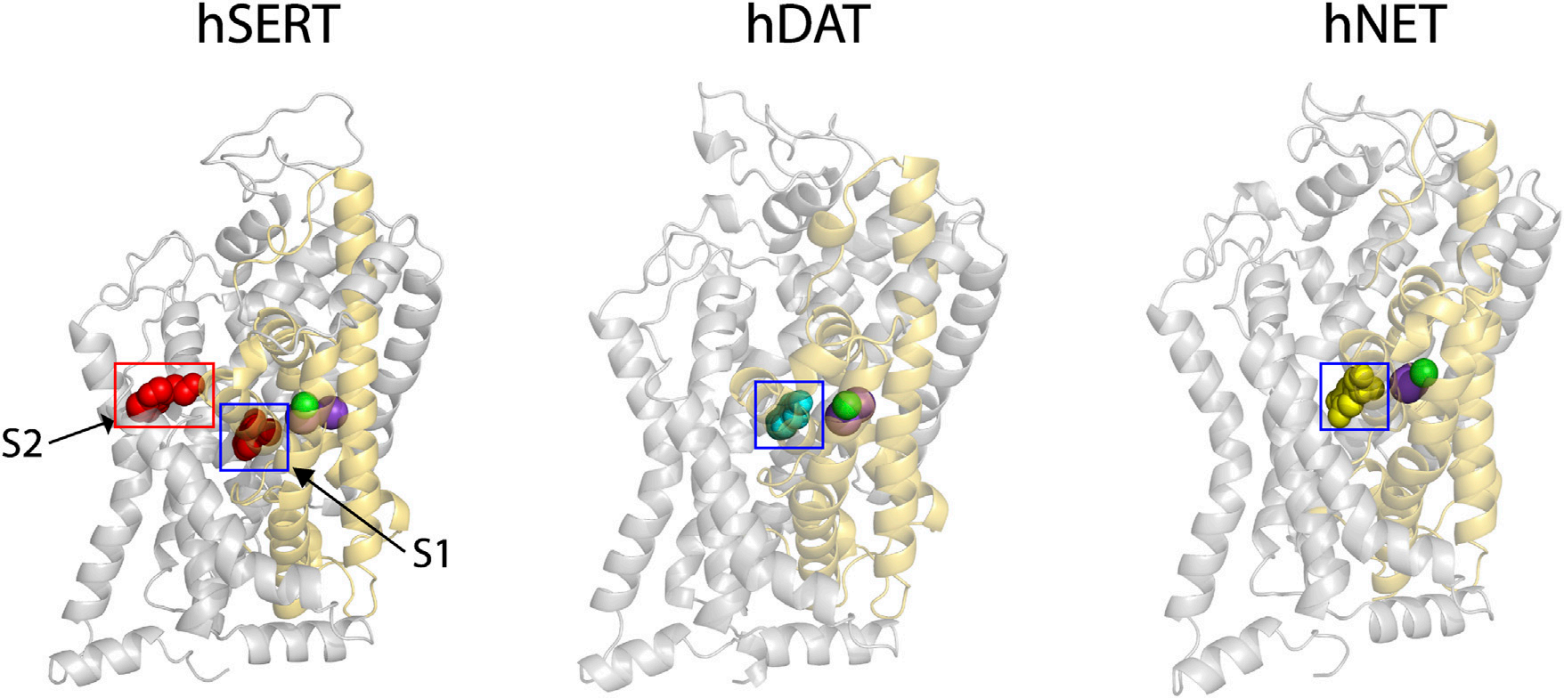
The cryo-EM structure of hSERT and homology models of hDAT and hNET. Two substrate (*red spheres*)-bound sites, the central site (S1, *blue square*) and allosteric site (S2, *red rectangle*), were localized in the cryo-EM structure of hSERT in an outward open conformation (PDB ID, 7LIA, *left*) (Yang and Gouaux, 2021). The homology models of hDAT (*middle*) and hNET (*right*) bound with the corresponding substrate in the S1 site (*cyan spheres* for dopamine and *yellow* for norepinephrine) were generated with Modeller based on an outward-facing structure of the *Drosophila melanogaster* DAT (2.89 Å resolution, PDB ID, 4XP1). Two Na^+^ (*purple spheres*) and one Cl^−^ (*green spheres*) ions are shown in the binding site of each transporter. The core and scaffold domains in each transporter are shown in golden and grey, respectively.

Our previous results showed that SAG strongly inhibited hSERT activity, but also weakly inhibited both hDAT and hNET (Huang et al., 2022). To explore the molecular mechanism of action of the herbal molecule by a structural approach, homology models for both hDAT and hNET were generated with Modeller based on an outward open structure of the *Drosophila* DAT (dDAT, PDB ID 4XP1). The sequence identity between the target and dDAT is 52% for hDAT or 55% for hNET, respectively, and therefore we expect that the Cα trace of either hDAT or hNET is accurate to within 1 Å of the native structure (Olivella et al., 2013). Na^+^ and Cl^−^ ions in the binding site were modeled on those observed in the dDAT template structure (Figure 1, purple spheres for Na^+^ and green for Cl^−^). The substrate dopamine or norepinephrine was also modeled into the central binding site (S1) of hDAT or hNET, respectively, based on the dopamine molecule observed in dDAT (Figure 1, cyan spheres for dopamine in hDAT and yellow spheres for norepinephrine in hNET).

Recent cryo-EM structures of hSERT in several conformational states have revealed an allosteric site (S2) formed by an aromatic pocket positioned in the scaffold domain in the extracellular vestibule (Yang and Gouaux, 2021). The substrate, 5-hydroxytryptamine (5-HT), was found to bind to the two sites, the central site (S1) and allosteric site (S2), in the hSERT structures (Figure 1, red spheres for 5-HT). Previous studies have demonstrated that the S1 site is essential for substrate binding, conformational conversion, and ion-coupled 5-HT transport (Rudnick, 2006; Forrest et al., 2008; Tao et al., 2009; Tavoulari et al., 2009; Andersen et al., 2014; Fenollar-Ferrer et al., 2014; Rudnick et al., 2014; Rudnick and Sandtner, 2019). In contrast, the allosteric site was speculated to provide 5-HT molecules to the S1 site once the transporter switches to the outward open conformation to rapidly trigger substrate transport (Yang and Gouaux, 2021). Given their structural and functional similarities, it is possible that all monoamine transporters have a similar allosteric site. For a comparison with hDAT or hNET, we selected the structure of hSERT bound with 5-HT in an outward open conformation for further docking study.

### Molecular docking of (-)-syringaresinol-4-O-β-D-apiofuranosyl-(1→2)-β-D-glucopyranoside to the allosteric site

**FIGURE 2:**
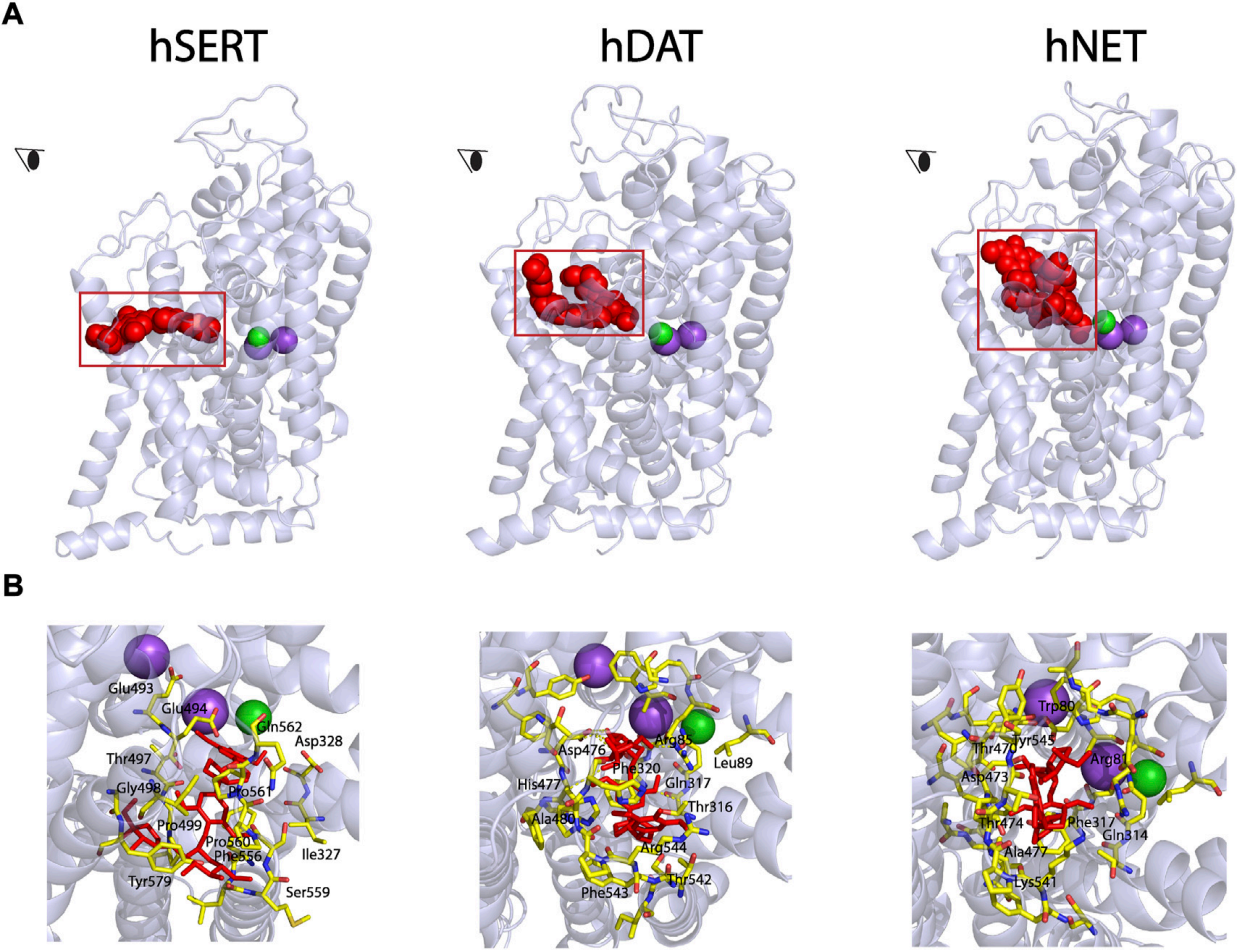
Molecular docking of SAG to the allosteric site in hSERT, hDAT, and hNET. **(A)** Overall views of hSERT-SAG (*left*), hDAT-SAG (*middle*), and hNET-SAG (*right*) complexes in cartoon representation. SAG (*red*) in the allosteric binding site was depicted as spheres. Purple and green spheres represents Na^+^ and Cl^−^ ions, respectively. The eye represents the angle views depicted in **(B)**. **(B)** Close-ups of SAG binding in the allosteric site. Residues that interact with SAG are annotated and shown in *yellow sticks*.

Our previous study has demonstrated that SAG noncompetitively inhibited SERT activity (Huang et al., 2022). To examine the possibility that SAG binds to the allosteric site, we conducted the molecular docking of SAG to hSERT, hDAT, and hNET on the hSERT structure and the hDAT and hNET models in an outward open conformation, respectively. As shown in Figure 2 (close-up in the bottom left), SAG molecule in the S2 site of hSERT is coordinated by the residues in TM1, TM6a, TM10, TM11, and TM12. The residues that interact with SAG include Gln111 in TM1, Ile327, Asp328 in TM6a, Glu493, Glu494, Thr497, Gly498, Pro499 in TM10, Phe556, Ser559, Pro560, Pro561, Gln562 in TM11, Tyr579, Thr583 in TM12, of which Asp328 and Ser559 form H-bonds with apiofuranosyl group of SAG. On the other hand, SAG adopts a bent conformation in the S2 site of both hDAT and hNET models (Figure 2, bottom middle and right). The SAG binding site in either the hDAT-SAG or hNET-SAG complex is formed by the residues from TM1, TM6a, TM10, and TM11, which includes Arg85, Leu89, Thr316, Gln317, Phe320, Asp476, His477, Ala480, Thr542, Phe543, and Lys544 in hDAT, or Trp80, Arg81, Gln314, Phe317, Thr470, Asp473, Thr474, Ala477, Lys541, and Tyr545 in hNET, respectively. More than half of the residues that are proposed to be involved in the SAG binding are identical between hDAT and hNET, but they significantly differ from those in hSERT, according to a structure-based alignment of the NSS transporters (Beuming et al., 2006).

### Influence of (-)-syringaresinol-4-O-β-D-apiofuranosyl-(1→2)-β-D-glucopyranoside on the cytoplasm-facing conformation in SERT

We previously investigated the influence of SAG on the outward-facing conformation in SERT by using accessibility measurement of a strategically positioned cysteine residue, Y107C, in the extracellular substrate permeation pathway (Huang et al., 2022). Our results showed that SAG decreased the accessibility of Y107C by inducing a conformational shift toward an outward-closed state of SERT, indicating SAG acted on SERT differently than a conventional antidepressant fluoxetine did. However, the previous study did not address the SAG influence on the cytoplasmic-facing conformation due to a technical difficulty. We have recently developed a novel approach for measuring ion- or ligand-induced conformational changes in the cytoplasmic permeation pathway in SERT, which allows us to determine the effect of SAG on the reactivity of a cysteine residue in the cytoplasmic permeation pathway with a cysteine reagent in digitonin-permeabilized cells (Li M. et al., 2022a).

We previously demonstrated that the cysteine residue in the cytoplasmic pathway, S277C, reacted with the cysteine reagent MTSEA more when the cytoplasmic pathway is open, and less when the pathway is closed (Rudnick, 2006). These measurements of the reactivity depend on the ability of the cysteine reagents such as MTSEA to inactivate ligand binding activity of SERT by an allosteric mechanism (Jacobs et al., 2007). We proposed that, by modifying a cysteine residue such as S277C in the cytoplasmic permeation pathway, MTSEA prevents the cytoplasmic pathway closing and the extracellular pathway opening, thus leading to the inactivation of ASP^+^ binding to SERT.

**FIGURE 3:**
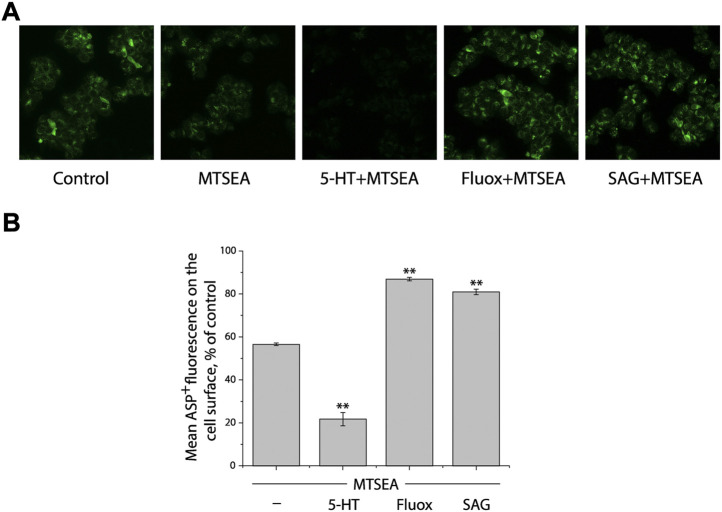
Influence of SAG on the cytoplasmic-facing conformation in SERT. **(A)** Representative images of ASP^+^ binding on the cell membrane of SERT mutant, S277C/X5C, after treatment without (control) or with 0.2 mM MTSEA in the absence (MTSEA alone) or presence of 10 μM 5-HT (5-HT + MTSEA), 10 μM fluoxetine (Fluox + MTSEA), or 10 μM SAG (SAG + MTSEA) in KRH buffer containing 25 μg/ml digitonin. The experiment was repeated twice more with similar results. **(B)** Quantitative analysis for ASP^+^ binding after treatment with the indicated drugs in the absence or presence of MTSEA, expressed as a percentage of that measured in the absence of MTSEA (control). In each experiment, at least ten cells were randomly selected for quantitative analysis. ASP^+^ fluorescence was counted and normalized to the cell membrane areas. All error bars represent the SEM (*n* = 3). Asterisks indicate statistically significant changes (** *p* < 0.01) in ASP^+^ fluorescence compared with the MTSEA alone using One-Way ANOVA.

We first determined a concentration of MTSEA (0.2 mM) that inactivated ∼50% of ASP^+^ binding in the cell plasma membrane of SERT mutant S277C in the presence of 25 μg/ml digitonin (Figures 3A,B, second image and first column from left, respectively). In the experiments to examine the effects of ligands on SERT conformation, we incubated cells stably expressing S277C with the indicated ligands and 0.2 mM MTSEA in the presence of 25 μg/ml digitonin. At the end of this incubation, cells were washed free of MTSEA and ligands into KRH buffer containing NaCl and ASP^+^. Altered ligand addition, therefore, was present only during the incubation with MTSEA and not during the ASP^+^ binding measurements. In this study, we measured the ability of ligands to influence the reactivity of S277C with MTSEA in the digitonin-permeabilized cells by confocal image analysis (Figure 3).

Substrate 5-HT, which stabilizes the cytoplasmic-facing conformation of SERT in the presence of both Na^+^ and Cl^−^ (Forrest et al., 2008), significantly increased the reactivity of S277C, promoting its inactivation by MTSEA (Figures 3A,B, third image and second column from left, respectively), consistent with our previous observation that 5-HT induced conformational conversion from outward-facing to inward-facing (Huang et al., 2022). In contrast, fluoxetine, a SSRI, markedly decreased the reactivity of S277C, protecting it from MTSEA inactivation (Figures 3A,B, second image and second column from right, respectively). On the other hand, SAG exhibited a potency to protect S277C from MTSEA inactivation, as compared to treatment with MTSEA alone (Figures 3A,B, first image and first column from right, respectively), indicating SAG influences the cytoplasmic-facing conformation in SERT by a mechanism similar with fluoxetine but different from 5-HT. Therefore, the results together with those from our previous observation demonstrate that SAG stabilizes a conformation of hSERT with both the extracellular and cytoplasmic pathways closed.

### Influence of the S1 and S2 mutations of human SERT on (-)-syringaresinol-4-O-β-D-apiofuranosyl-(1→2)-β-D-glucopyranoside inhibition potency

**FIGURE 4:**
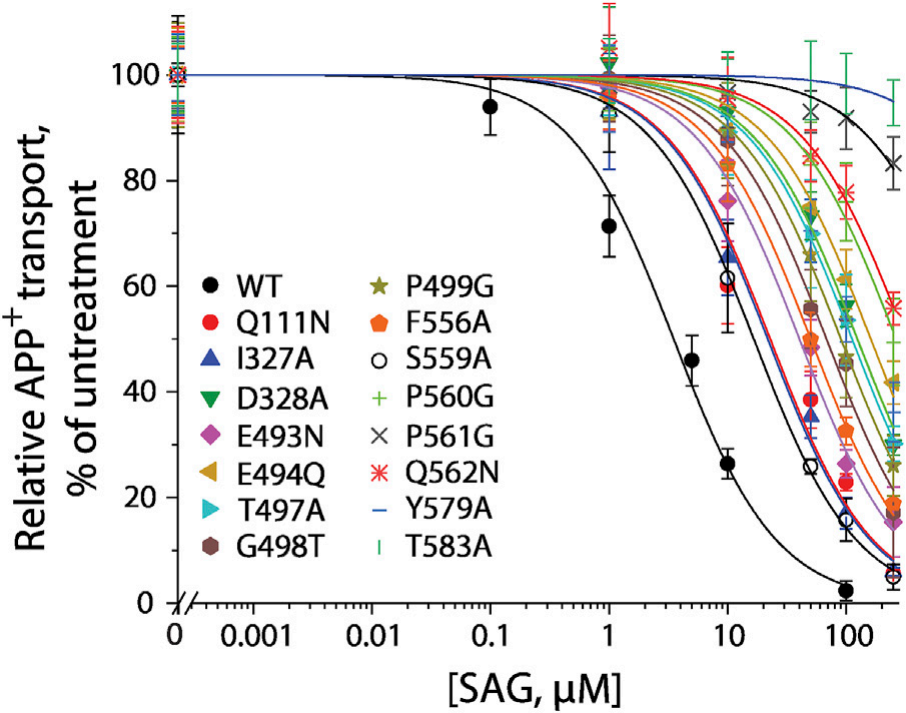
SAG inhibition of APP^+^ transport by hSERT and its S2 site mutants. APP^+^ transport into the cells expressing hSERT or its mutants was measured over the indicated range of SAG concentrations as described under “Materials and methods”. Nonspecific APP^+^ transport was measured in the presence of 10 μM fluoxetine and subtracted to give the values of APP^+^ transport. Graph shows representative experiments for SAG inhibition of hSERT and its mutants, with APP^+^ transport expressed as a percentage of that measured in the absence of SAG. All error bars represent SDs from at least ten measurements. Experiments were repeated twice more with similar results.

According to our molecular docking, we generated 15 mutants of the allosteric site in hSERT by site-directed mutagenesis and performed transport assays to examine their effects on SAG inhibition of APP^+^ transport by hSERT. All mutants were functional for APP^+^ transport (Table 1). Compared to WT-hSERT, transport activities of five mutants, E494Q, P560G, P561G, Q562N, and T583A were 1.4–2.0 folds higher, whereas three mutants, E493N, P499G, and F556A showed 25–38% relative transport activities of WT. The rest of mutants showed comparable activities to WT-hSERT. Figure 4 shows SAG concentration-dependent inhibition of APP^+^ uptake in WT-hSERT and its S2 mutants. The IC50 values for SAG were estimated based on their inhibition curves (Table 1). SAG inhibited WT-hSERT transport activity with an IC50 value of 2.54 ± 0.13 μM. By comparison, the IC50 values in all the S2 mutants tested were significantly increased. SAG inhibited APP^+^ transport in 13 of 15 mutants with more than 10-fold increased IC50 values, compared with that in WT; of those, four mutants, P560G, P561G, Q562N, and T583A, were inhibited by SAG with IC50 values approximate 100 or more folds higher than that in WT. These results indicate that replacement of the residues in the allosteric site of hSERT leads to dramatical reduction in SAG inhibition potency.

**TABLE 1:** Inhibition potency of SAG for WT and mutants of hSERT. APP^+^ transport or ASP^+^ binding was performed on intact or digitonin-permeabilized HeLa cells transiently expressing WT or allosteric and orthosteric mutants of hSERT as described under “Materials and methods”. For APP^+^ transport, cells were incubated with 2 μM APP^+^ for 5 min. SAG was added 5 min prior to APP^+^ to obtain equilibrium. IC50 values were calculated from non-linear regression analysis of APP^+^ uptake. The APP^+^ uptake rate without SAG addition for WT-hSERT was 43.32 ± 1.78 mean AFU/min. For ASP^+^ binding, SAG was added in the presence of 25 μg/ml digitonin 5 min prior to ASP^+^ addition. The permeabilized cells were incubated with 10 μM ASP^+^ for 5 min. The ASP^+^ binding without SAG addition for WT-hSERT was 20.89 ± 0.46 mean AFU.

hSERT	Mutating site	Transport activity (% of WT)	SAG IC50 for transport (μM)	SAG IC50 for binding (μM)
WT		100	2.54 ± 0.13	7.88 ± 0.35
Q111N		76.4 ± 1.8*	19.1 ± 2.2*	nd
I327A		86.3 ± 2.9	25.5 ± 1.2*	48.3 ± 4.6*
D328A		73.4 ± 2.1*	113 ± 5**	nd
E493N		29.2 ± 5.2**	53.2 ± 6.6**	nd
E494Q		164 ± 14	153 ± 2***	192 ± 16***
T497A		102 ± 2	111 ± 2***	152 ± 12***
G498T	S2	70.2 ± 1.3*	69.2 ± 3.3**	nd
P499G		37.0 ± 1.9**	88.1 ± 2.8**	nd
F556A		25.9 ± 0.3**	41.0 ± 6.5*	nd
S559A		69.5 ± 2.4*	20.2 ± 1.9*	126 ± 8.1**
P560G		173 ± 3**	243 ± 10***	324 ± 28***
P561G		201 ± 4**	>1000***	> 1000***
Q562N		141 ± 9	289 ± 11***	450 ± 23***
Y579A		81.7 ± 2.4	127 ± 3***	nd
T583A		202 ± 5**	>1000***	nd
Y95F		42.2 ± 1.3**	4.45 ± 0.17	nd
I172F	S1	53.6 ± 2.1**	12.52 ± 0.97*	nd
S438T		73.3 ± 3.0*	10.28 ± 0.49*	nd

Data in Table 1 were shown as mean ± SEM from three experiments. Asterisks indicate significant difference in APP^+^ transport activity or ASP^+^ binding inhibition potency compared with WT-hSERT, respectively (* *p* < 0.05; ** *p* < 0.01; *** *p* < 0.001, One-Way ANOVA). nd, not determined.

To examine if the S1 site also influences SAG inhibition of APP^+^ transport, we mutated several S1 residues, such as Tyr95, Ile172, and Ser438 that have been demonstrated to directly participate in antidepressant binding (Coleman et al., 2016). As shown in Table 1, substitution of Tyr95 with Phe had little effect on SAG inhibition potency, whereas replacement of Ile172 with Phe or Ser438 with Thr resulted in a slightly decreased SAG inhibition potency with an IC50 value less than 5 folds higher than that in WT. Because these S1 mutants have been showed to possess *K*_*m*_ values for substrate 5-HT lower than that in WT (Andersen et al., 2010), we assumed that the small decrease in SAG inhibition potency is probably due to an increase in substrate binding affinity caused by the mutation rather than an alteration in SAG binding in the S2 site. By comparison with the dramatic effects of the S1 site residues on the binding affinity of SSRIs, such as citalopram and fluoxetine (Andersen et al., 2009; Andersen et al., 2010; Andersen et al., 2014; Coleman et al., 2016; Rannversson et al., 2016; Plenge et al., 2020), our results suggest that SAG does not interfere with the S1 site.

Furthermore, we selected several S2 mutants to investigate their effects on ligand binding. Taking advantage of ASP^+^ binding ability with micromolar affinity to SERT in the cell membrane (Oz et al., 2010), we performed fluorescence image analysis to measure SAG inhibition of ASP^+^ binding in digitonin-permeabilized cells. As shown in Table 1, the representative S2 mutants showed markedly increased IC50 values for SAG inhibition of ASP^+^ binding compared with that in WT, consistent with their influences on SAG inhibition of APP^+^ transport. These results indicate that the S2 mutations result in significant decreases in SAG binding affinity.

### Influence of the S2 mutations of human DAT and human NET on (-)-syringaresinol-4-O-β-D-apiofuranosyl-(1→2)-β-D-glucopyranoside inhibition potency

It has been reported that the monoamine transporters, SERT, DAT, and NET, all uptake both APP^+^ and ASP^+^ (Schwartz et al., 2003; Mason et al., 2005; Schwartz et al., 2005; Bolan et al., 2007; Haunso and Buchanan, 2007; Zapata et al., 2007; Jorgensen et al., 2008; Oz et al., 2010; Solis et al., 2012; Wilson et al., 2014; Bhat et al., 2021), ASP^+^, however, exhibits binding-associated fluorescence in the plasma membrane, which easily obscures transport activity (Oz et al., 2010; Bhat et al., 2021). Our previous experiments indicated that hSERT transports APP^+^ with a *K*_*m*_ value of 2.63 ± 0.16 μM. In this study, we performed kinetic analysis for both hDAT and hNET. The *K*_*m*_ values for APP^+^ in hDAT and hNET were 2.89 ± 0.48 μM and 1.56 ± 0.22 μM, respectively. The similarity in the substrate binding affinity allow us to investigate the SAG inhibition potency on the three transporters, hSERT, hDAT and hNET, by using the same substrate, APP^+^.

**FIGURE 5:**
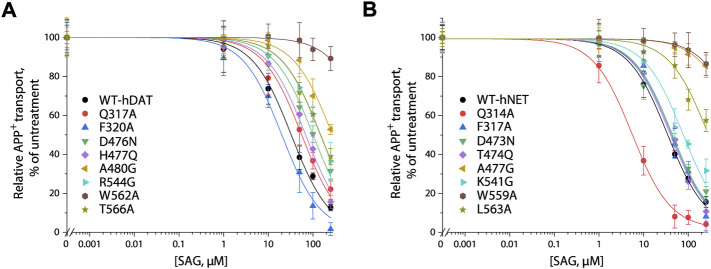
SAG inhibition of APP^+^ transport by hDAT, hNET and their S2 site mutants. APP^+^ transport into the cells expressing hDAT, hNET, or their mutants was measured over the indicated range of SAG concentrations as described under “Materials and methods”. Nonspecific APP^+^ transport was measured in the presence of 10 μM GBR12909, or 10 μM desipramine and subtracted to give the values of APP^+^ transport by hDAT or hNET, respectively. Graphs show representative experiments for SAG inhibition of WT and mutants of hDAT **(A)**, WT and mutants of hNET **(B)** with APP^+^ transport expressed as a percentage of that measured in the absence of SAG. All error bars represent SDs from at least ten measurements. Experiments were repeated twice more with similar results.

We generated 8 mutants of the S2 site in either hDAT or hNET, respectively, based on our docking of SAG to the transporter models and experimental results from SAG inhibition of hSERT transport activity. The corresponding residues that were shown to exert profound effects on SAG inhibition of hSERT transport or binding activity, were mutated in both hDAT and hNET, respectively. Several residues that are unique for the SAG binding in hDAT and hNET models were also selected for site-directed mutagenesis. As shown in Table 2, most of the mutants of both hDAT and hNET showed comparable transport activities with WT transporters, except for two hDAT mutants, F320A and A480G, and two hNET mutants, Q314A and F317A, which were functional for APP^+^ transport with activities less than 50% of WT. Figure 5 shows SAG inhibition of APP^+^ transport by WT and mutants of hDAT (Figure 5A) and hNET (Figure 5B). By comparison with WT-hDAT, SAG IC50 values in 6 of 8 mutants of hDAT were increased; of those, five mutants, D476N, H477Q, A480G, R544G, and T566A showed less than 10-fold increases in SAG IC50 values, whereas W562A exerted a more than 30-fold increase in SAG IC50. On the other hand, SAG IC50 value in one mutant, F320A, was decreased by approximate 50% compared to that in WT-hDAT. To further validate the association of SAG with the S2 site in hDAT, we mutated several S1 residues, such as Phe76, Val152, and Ser422, which have been previously shown to play critical roles in recognition of cocaine and benztropine binding (Beuming et al., 2008; Bisgaard et al., 2011). As shown in Table 2, replacement of these S1 residues had little effect on SAG inhibition potency for APP^+^ transport, supporting that SAG binds to the S2 site in hDAT.

**TABLE 2:** Inhibition potency of SAG for WT and mutants of hDAT and hNET. APP^+^ transport was performed on intact HeLa cells transiently expressing WT or allosteric and orthosteric mutants of hDAT and hNET as described under Table 1. The APP^+^ uptake rate without SAG addition for WT-hDAT and WT-hNET were 36.23 ± 1.91 mean AFU/min and 33.91 ± 2.23 mean AFU/min, respectively.

hDAT	Mutating site	Transport activity (% of WT)	SAG IC50 (μM)	hNET	Transport activity (% of WT)	SAG IC50 (μM)
WT		100	31.9 ± 2.8	WT	100	40.1 ± 2.8
Q317A		100 ± 3	51.3 ± 3.1	Q314A	20.2 ± 0.4**	4.37 ± 1.06**
F320A		30.5 ± 4.6*	16.8 ± 0.7*	F317A	17.2 ± 0.5**	36.7 ± 7.6
D476N		66.1 ± 2.8*	93.8 ± 3.9**	D473N	124 ± 4	42.7 ± 4.7
H477Q	S2	110 ± 2	72.4 ± 4.8*	T474Q	103 ± 5	49.3 ± 7.9
A480G		47.6 ± 1.3*	239 ± 14***	A477G	138 ± 5*	>1000***
R544G		51.4 ± 1.5*	150 ± 4***	K541G	78.5 ± 2.6*	79.8 ± 3.3*
W562A		71.2 ± 2.2*	>1000***	W559A	129 ± 5*	>1000***
T566A		122 ± 5	136 ± 6**	L563A	133 ± 6*	304 ± 12***
F76V		22.5 ± 1.3	18.2 ± 0.8			
V152F	S1	38.2 ± 2.3	26.6 ± 2.8			
S422T		52.8 ± 1.8	24.5 ± 1.4			

Data were shown as mean ± SEM from three experiments. Asterisks indicate significant difference in APP^+^ transport activity or ASP^+^ binding inhibition potency compared with WT-hDAT or WT-hNET, respectively (* *p* < 0.05; ** *p* < 0.01; *** *p* < 0.001, One-Way ANOVA).

In comparison with hDAT, mutating the corresponding residues in hNET resulted in a small but significant difference in SAG inhibition of transport activity (Figure 5B and Table 2). Three mutants of hNET, F317A, D473N, and T474Q, showed little change in SAG inhibition potency compared to WT-hNET. On the other hand, SAG IC50 values were increased by less than 10 folds in two mutants, K541G and L563A, but more than 25 folds in other two mutants, A477G and L559A, respectively, compared to that in WT-hNET. Q314A was unique because its IC50 value for SAG inhibition was decreased by approximate 10 folds. These results indicate that substitution of the S2 residues in either hDAT or hNET led to significant effects on SAG inhibition, however, the influence on the two transporters, hDAT and hNET, was somewhat different.

### The S2 binding sites for vilazodone and (-)-syringaresinol-4-O-β-D-apiofuranosyl-(1→2)-β-D-glucopyranoside in human SERT overlap

**FIGURE 6:**
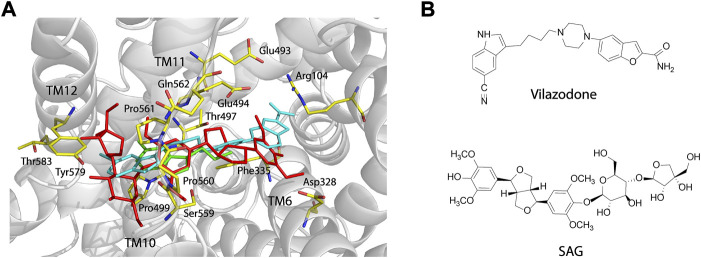
Comparison of the vilazodone binding pose with the binding poses for SAG and 5-HT within the S2 site and structures of vilazodone and SAG. **(A)** Comparison of the binding poses for vilazodone, SAG, and 5-HT within the S2 site. The main chain position of hSERT in the hSERT-imipramine-vilazodone complex structure (PDB ID, 7lWD) is shown in *grey*. Vilazodone, 5-HT (PDB ID, 7LIA), and SAG are shown in *cyan*, *green*, and *red sticks*, respectively. **(B)** Structures of vilazodone and SAG.

Vilazodone, an approved antidepressant drug, also acts on 5-HT_1A_ receptor as a partial agonist (Dawson and Watson, 2009). A recent cryo-EM structure of SERT-imipramine-vilazodone complex has revealed that vilazodone binds to the S2 site that includes an aromatic pocket formed by TM10, 11, and 12 (Plenge et al., 2021). To compare the binding of SAG (red sticks, Figure 6) with vilazodone (cyan sticks) and the substrate 5-HT (green sticks) in the S2 site, we superimposed both SAG and 5-HT onto the structure of SERT bound with vilazodone. As shown in Figure 6, both the allosteric inhibitors expand the boundaries of the extracellular vestibule where the substrate 5-HT binds with. The bulky backbones of the two compounds adopt nearly linear binding poses across the extracellular vestibule. The binding sites for vilazodone and SAG are mainly formed by the residues in TM10, TM11, and TM12 and the side chains of some residues that interact with vilazodone are identical with those in SAG binding, such as Phe335, Glu494, Thr497, Gly498, Pro499, Ser559, Pro560, Pro561, and Gln562. The key differences between vilazodone and SAG binding involve interactions between the distal end moieties of the inhibitor molecules and their interacting residues. The glucopyranoside and apiofuranosyl moiety of SAG extend toward TM12, interacting with the side chains of Tyr579 and Thr583. In addition, the dimethoxyphenyl moiety in another distal end of SAG stretches toward TM6, interacting with Asp328 by an H-bond, whereas the benzofuran carboxamide moiety of vilazodone protrudes into a subsite near the extracellular salt-bridge formed by Arg104 and Glu493.

## Discussion

The SSRIs targeting SERT have been demonstrated to bind with high affinity to the central binding site and thus to competitively inhibit 5-HT transport, they, however, have many shortcomings, such as slow onset, low efficacy, and serious adverse effects, supporting the development of novel agents that exert a variety of pharmacological actions (Coleman et al., 2020; Plenge et al., 2021). An allosteric site, as the second binding site where substrate or ligand binding modulates transport function, has been mapped to be located in the extracellular vestibule connected to the central binding site in SERT by various biochemical approaches, including photoaffinity labeling, mutagenesis, or radiolabeled ligand binding (Plenge et al., 2012; Niello et al., 2020). Afterwards, atomic force microscopy experiments and the crystal and cryo-EM structures of SERT confirmed the presence of the S2 site in SERT (Coleman et al., 2016; Zhu et al., 2016; Zhu et al., 2020; Yang and Gouaux, 2021). The allosteric inhibitory effect has been proposed to be due to steric hindrance of the extracellular permeation pathway for substrate or ligand binding in the S1 site (Rannversson et al., 2016; Niello et al., 2020). A similar site has also been proposed in DAT and NET based on the studies of the dissociation of radiolabeled ligands (Plenge and Mellerup, 1997; Khelashvili et al., 2013; Stockner et al., 2013; Niello et al., 2019; Zhu et al., 2020). These studies open the possibility of developing novel therapeutic agents by targeting the S2 site. Two agents, Lu AF60097 and vilazodone, have recently been demonstrated to bind to the S2 site in SERT with high affinity by pharmacological and structural approaches, respectively (Plenge et al., 2020; Plenge et al., 2021).

This work validated the molecular mechanism of SAG inhibition through its binding to the allosteric S2 site and blocking of conformational conversion, thus uncompetitively inhibiting hSERT transport activity by using the molecular docking and mutagenesis approaches. The evidence presented here support the proposal that SAG directly binds to the S2 site in hSERT. Our results showed that 1) SAG associates with the S2 site of hSERT in the molecular docking; 2) mutating residues in the S2 site but not the S1 site leads to dramatical reductions in SAG inhibition of hSERT transport and ligand binding; 3) SAG stabilizes a conformation of hSERT with both the extracellular and cytoplasmic pathways closed (Huang et al., 2022). Taken together, our computational and biochemical results indicate that the herbal molecule, SAG, acts on hSERT by a underlying mechanism of action different from that of SSRIs.

Although SAG strongly inhibited hSERT activity, the herbal molecule also exerted an inhibitory effect on both hDAT and hNET. Its IC50 values in hDAT and hNET were 11 or 16 folds higher than that in hSERT, indicating that SAG is slightly selective in antagonizing hSERT over hDAT or hNET. The higher selectivity of SAG in inhibiting hSERT was also evidenced by our experimental results of site-directed mutagenesis of the S2 site in these monoamine transporters. Replacement of the S2 residues in hSERT, such as D328A, E494Q, T497A, P560G, P561G, Q562N, Y579A, and T583A, led to profound reductions in SAG inhibition potency by more than 40 folds (Table 1), whereas the similar substitution of the corresponding residues, such as D476N, H477Q for hDAT and D473N, T474Q for hNET, decreased SAG inhibition potency by less than 3 folds (Table 2). On the other hand, replacing the unique residues in either hDAT or hNET, such as A480G for hDAT and A477G for hNET, resulted in an increase in SAG IC50 values by 7–25 folds compared to those in the WT transporters. These pharmacological data suggested that SAG binding in hSERT was distinct from that in either hDAT or hNET, consistent with our molecular docking of SAG to the allosteric S2 site, in which SAG presents at an extended conformation in hSERT different from SAG at a bent conformation in either hDAT or hNET.

**FIGURE 7:**
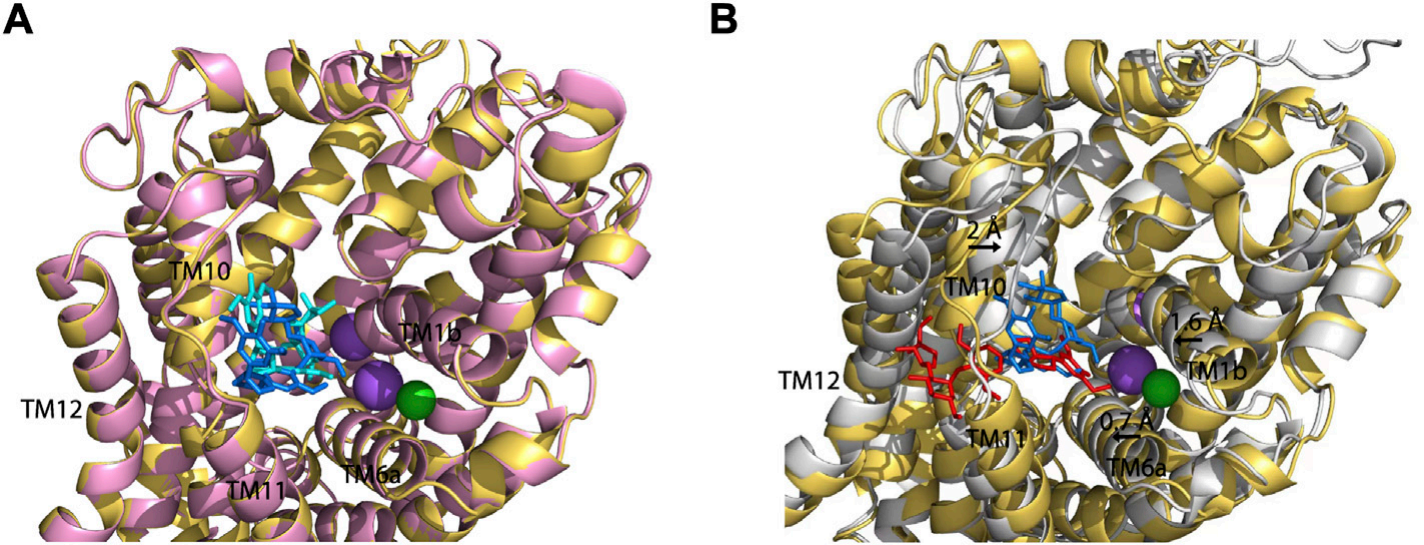
Superposition on the allosteric sites of hDAT-SAG, hNET-SAG and hSERT-SAG. **(A)** Superposition on the allosteric sites of hDAT-SAG (*golden*, SAG in *blue*) and hNET-SAG (*pink*, SAG in *cyan*) in outward open states. Na^+^ (*purple*) and Cl^−^ (*green*) ions in the hDAT model were only shown. **(B)** Superposition on the allosteric sites of hDAT-SAG (*golden*, SAG in *blue*) and hSERT (*grey*, SAG in *red*) in outward open states. Na^+^ (*purple*) and Cl^−^ (*green*) ions in the SERT structure were only shown.

To compare SAG binding in all three monoamine transporters, we superimposed the molecular docking models of the hSERT-SAG, the hDAT-SAG, and the hNET-SAG complexes (Figure 7). hDAT and hNET superimposed almost seamlessly in our homology models, possibly due to a 67% sequence identity between the two transporters (Figure 7A). SAG adopts a bent conformation with a slight difference in coordination with the residues in the S2 site between hDAT and hNET models, consistent with our biochemical results that demonstrated a small difference in the influence of S2 mutations on SAG inhibition between hDAT and hNET. We speculated that the configuration of SAG in the S2 site is account for the difference in the influence of S2 mutations on its inhibition potency between hDAT and hNET. On the other hand, hSERT-SAG complex displays a configuration with a reduced allosteric cavity that is clearly narrower than that seen in either hDAT or hNET. The movement of TM1b, TM6a, and TM10 toward the center of the allosteric cavity in hSERT results in a compact site where the spatial constraints could not allow SAG to bind at a bent conformation (Figure 7B). Strikingly, a cluster of residues including Ser559, Pro560, Pro561, and Gln562 in TM11 of hSERT that is unique among the monoamine transporters was observed to directly interact with the glucopyranoside and apiofuranosyl groups through H-bond, hydrophilic and hydrophobic interactions in our docking model. The observation was supported by our mutagenesis results that showed a dramatic decrease in SAG inhibition potency by substituting these residues. The unique interaction between SAG and the residue cluster in TM11, in turn, might allow SAG to expand the boundary of the extracellular vestibule toward TM12 in hSERT, as vilazodone did in the allosteric S2 site (Plenge et al., 2021). These observations suggest that the specific interaction provides a distinct coordination for SAG binding in the S2 site of hSERT. Thus, we assume that the different configuration in the allosteric S2 site between hSERT and either hDAT or hNET determines the specificity of SAG in inhibiting monoamine transporters.

Taken together, we present here that the herbal molecule, SAG, binds to the allosteric site of monoamine transporters through the various interactions with various residues, which opens for the possibility of developing novel antidepressant drugs with distinct pharmacodynamic profiles and specificities. Although SAG strongly inhibits monoamine transporters and exerts a specificity for hSERT, its inhibition potency and selectivity are lower than those of SSRIs. It is notable that the allosteric site of monoamine transporters has been underexplored in structure-based drug design (Garibsingh and Schlessinger, 2019). We anticipate that further improvement of inhibition potency and specificity of SAG by structural modification could promote discovery of antidepressant and anxiolytic drugs with a novel underlying mechanism of action.

## Data Availability

The original contributions presented in the study are included in the article/Supplementary Materials; further inquiries can be directed to the corresponding author.
